# Supplementary material for: Authors submission guidelines, a survey of pediatric dentistry journals regarding ethical issues
Source: PLoS One. 2022 Jan 19;17(1):e0261881. doi: 10.1371/journal.pone.0261881 (PMC8769321; doi:10.1371/journal.pone.0261881)
Supplement: S1 Appendix — (DOCX) [file pone.0261881.s001.docx]

| S. NO. | JOURNAL NAME | FOR AUTHORSHIP (ICMJE) | ETHICAL APPROVAL | INFORMED CONSENT | ANIMAL WELFARE | COI | PUBLICATION ETHICS | research misconduct | complaints about Authors | complaints about Reviewers | complaints about Editorial team | Authorship disputes | Reporting Criteria’s for Studies | Availability of raw data | Copyright Issues | Impact factor JCR 2020 |
| --- | --- | --- | --- | --- | --- | --- | --- | --- | --- | --- | --- | --- | --- | --- | --- | --- |
| 1 | [European archives of paediatric dentistry](https://journals.indexcopernicus.com/search/details?id=54398) | YES | YES | YES | YES | YES | YES | YES | YES | NO | YES | YES | YES | YES | YES | NA |
| 2 | European journal of paediatric dentistry | NO | YES | YES | NO | NO | NO | NO | NO | NO | YES | NO | NO | NO | NO | **2.231** |
| 3 | International Journal of Clinical Pediatric Dentistry | NO | YES | YES | NO | YES | NO | NO | NO | NO | YES | NO | NO | NO | YES | NA |
| 4 | [International Journal of Paediatric Dentistry](https://journals.indexcopernicus.com/search/details?id=29601) | NO | YES | YES | YES | YES | YES | YES | YES | NO | YES | NO | YES | YES | YES | 3.455 |
| 5 | [International Journal of Pedodontic Rehabilitation](https://doaj.org/toc/2468-8940) | YES | YES | YES | NO | YES | NO | NO | NO | NO | YES | NO | YES | NO | YES | NA |
| 6 | Interventions in Pediatric Dentistry Open Access Journal | NO | NO | NO | NO | YES | NO | NO | NO | NO | YES | NO | NO | NO | NO | NA |
| 7 | Journal of Clinical Pediatric Dentistry | NO | NO | YES | NO | YES | NO | NO | NO | NO | NO | NO | NO | NO | YES | 1.065 |
| 8 | JOURNAL OF DENTISTRY FOR CHILDREN | YES | YES | YES | YES | YES | YES | YES | YES | YES | YES | YES | YES | YES | YES | NA |
| 9 | [Journal of Indian Society of Pedodontics and Preventive Dentistry](https://journals.indexcopernicus.com/search/details?id=35759) | YES | YES | YES | NO | YES | NO | NO | NO | NO | YES | NO | NO | NO | YES | NA |
| 10 | Journal of South Asian Association of Pediatric Dentistry | YES | YES | YES | YES | YES | YES | NO | NO | NO | NO | NO | YES | NO | YES | NA |
| 11 | [Odontologia Pediat̕rica](https://www.worldcat.org/title/odontologa-peditrica/oclc/921230733&referer=brief_results) | NO | NO | NO | NO | YES | NO | NO | NO | NO | NO | NO | NO | NO | NO | NA |
| 12 | [Paidodontía](https://www.worldcat.org/title/paidodontia-trimeniaia-ekdose-tes-hellenikes-paidodontikes-hetaireias-quarterly-publication-of-the-hellenic-society-of-paediatric-dentistry/oclc/56576606&referer=brief_results) | NO | NO | NO | NO | YES | NO | NO | NO | NO | NO | NO | NO | NO | YES | NA |
| 13 | PEDIATRIC DENTAL JOURNAL | YES | YES | YES | YES | YES | YES | YES | YES | NO | NO | YES | YES | YES | YES | NA |
| 14 | Pediatric Dentistry | YES | YES | YES | YES | YES | YES | YES | YES | YES | YES | YES | YES | YES | YES | 1.874 |
| 15 | [Pesquisa Brasileira em Odontopediatria e Clínica Integrada (Brazilian Research in Pediatric Dentistry](https://doaj.org/toc/1983-4632) | NO | YES | NO | YES | NO | NO | NO | NO | NO | NO | NO | NO | NO | YES | NA |
| 16 | [Revista Latinoamericana de ortodoncia y odontopediatría.](https://www.worldcat.org/title/revista-latinoamericana-de-ortodoncia-y-odontopediatria/oclc/261341392&referer=brief_results) | YES | YES | YES | YES | YES | YES | YES | YES | YES | YES | YES | YES | YES | YES | NA |
| 17 | [Shōni shikagaku zasshi. The Japanese journal of pedodontics](https://www.ncbi.nlm.nih.gov/nlmcatalog/136612) | YES | YES | YES | YES | YES | YES | YES | YES | NO | NO | YES | YES | YES | YES | NA |
| 18 | [Taehan Soa Chʻikwa Hakhoe chi = Journal of the Korean Academy of Pedodontics.](https://www.ncbi.nlm.nih.gov/nlmcatalog/9814921) | NO | YES | YES | YES | YES | NO | NO | NO | NO | NO | NO | NO | NO | NO | NA |
