# Supplementary material for: Authors submission guidelines, a survey of pediatric dentistry journals regarding ethical issues
Source: PLoS One. 2022 Jan 19;17(1):e0261881. doi: 10.1371/journal.pone.0261881 (PMC8769321; doi:10.1371/journal.pone.0261881)
Supplement: Attachment — Submitted filename: Review PONE-D-21-18572.docx [file pone.0261881.s002.docx]

**REVIEW OF THE PONE-D-21-18572 PAPER WITH TITLE:**

**“Survey of Submission Guidelines for Authors on Ethical Issues in Pediatric Dentistry Journals”**

**SECTIONS**

**According to the submission guidelines of the journal, the major sections of the article should be as follows: ABSTRACT, INTRODUCTION, MATERIALS AND METHODS, RESULTS, DISCUSSION, CONCLUSIONS. Τhe “MATERIALS AND METHODS” section has been omitted. Please add this major section and use sub-sections for search strategy e.t.c. Please use the same format for the sections of the same level, as presented in the sample manuscript body included in the Journal Submission Guidelines.**

**ABSTRACT**

- **The “Background” is not necessary according to the Journal Guidelines in the Abstract Session and it can be omitted.**
- **The Objective of the study must be clearly reported in a separate subsection.**
- **The 3 subsections of Settings and Design, Materials and Methods and Statistical Analysis can be included all in a single subsection “Materials and Methods.**

**INTRODUCTION**

**In this section, the significance of the present study and the reason why the problem addressed is important are well presented. However, no reference is made to the existing literature or whether similar studies have been carried out. Ιt is not clear what is new that this study offers in the existing literature. Nor is the purpose of the study clearly stated. This information could be included in two additional separate paragraphs.**

**MATERIALS AND METHODS**

- **The inclusion and exclusion criteria of the Journals could be presented more clearly.**
- **Was the authors' blindness to the Journal somehow ensured? If not, it must be mentioned.**
- **The statistical analysis performed should be reported at the end of this section in a separate paragraph.**

**RESULTS**

- **Concerning the search results, a flow chart would provide more information and be more explanatory.**
- **The results should be presented by absolute number too and not only by percentages.**
- **Table 1 or Table 2 could be presented as a bar graph instead of a table for better representation of the results graphically.**
- **In the text, there is no need to mention the percentages of compliant Journals for all 14 principles, as these are shown in the table. However, the emphasis should be given mainly to the most or least frequent ethical principles not mentioned in the Pediatric Dentistry Journals.**

**DISCUSSION**

- **This section is well written, and all aspects are analyzed correctly.**
- **In the second paragraph, reference citation “7” is repeated and a citation is missing at the end of this paragraph.**

**CONCLUSIONS**

- **Line 259 🡪 Please start with a new sentence “…Ethical issues…”**
- **It would be preferable and more readable if you could present the conclusions using bullets instead of a single paragraph.**

**TABLES**

- **Please use the same format for table titles, as follows: “Table 1. This is the Table 1 Title.”**

**REFERENCES**

- **Please cite references in brackets (for example, “[1]” or “[2-5]” or “[3,7,9]”).**
- **There is inconsistency in the references. Firstly, references with more than six authors should list the first six author names, followed by “et al.” Moreover, all references should follow exactly the same style, as the following example:**

**“Doe J, Data A, van Stats J, Testperson M, Ribosome D Jr, 94  McBio GHT, et al. This is the article title. PLoS ONE. 2017;12(12):e00000. doi: 10.1371/journal.pone.0000000”**
