# Supplementary material for: Authors submission guidelines, a survey of pediatric dentistry journals regarding ethical issues
Source: PLoS One. 2022 Jan 19;17(1):e0261881. doi: 10.1371/journal.pone.0261881 (PMC8769321; doi:10.1371/journal.pone.0261881)
Supplement: Attachment — Submitted filename: Response to Reviewers.docx [file pone.0261881.s003.docx]

| REVIEWER’S COMMENT | AUTHOR’S RESPONSE | Changes Done |
| --- | --- | --- |
| SECTIONS  According to the submission guidelines of the journal, the major sections of the article should be as follows: ABSTRACT, INTRODUCTION, MATERIALS AND METHODS, RESULTS, DISCUSSION, CONCLUSIONS. Τhe “MATERIALS AND METHODS” section has been omitted. Please add this major section and use sub-sections for search strategy e.t.c. Please use the same format for the sections of the same level, as presented in the sample manuscript body included in the Journal Submission Guidelines. | Appropriate sections have been mentioned with subsections in the Materials and Methods section. | Abstract- line 17  Introduction- line 36  Aims & objectives - line 73  Materials and methods- line 75  Results- line 144  Discussion- line 183  Conclusions- line 268 |
| ABSTRACT  The “Background” is not necessary according to the Journal Guidelines in the Abstract Session and it can be omitted. The Objective of the study must be clearly reported in a separate subsection. The 3 subsections of Settings and Design, Materials and Methods and Statistical Analysis can be included all in a single subsection “Materials and Methods. | Headings in the abstract modified | Changes done lines 17 to 34 |
| INTRODUCTION  In this section, the significance of the present study and the reason why the problem addressed is important are well presented. However, no reference is made to the existing literature or whether similar studies have been carried out. Ιt is not clear what is new that this study offers in the existing literature. Nor is the purpose of the study clearly stated. This information could be included in two additional separate paragraphs. | The reason to conduct this study has been highlighted in the last para of introduction. Reference study mentioned | Changes from lines 66 to 74 |
| MATERIALS AND METHODS   - The inclusion and exclusion criteria of the Journals could be presented more clearly. - Was the authors' blindness to the Journal somehow ensured?If not, it must be mentioned. - The statistical analysis performed should be reported at the end of this section in a separate paragraph. | Mentioned  Authors could not be blinded due to the design of study  Mentioned | Lines 76-90  Lines 102-103  Lines 142-143 |
| RESULTS   - Concerning the search results, a flow chart would provide more information and be more explanatory. - The results should be presented by absolute number too and not only by percentages. - Table 1 or Table 2 could be presented as a bar graph instead of a table for better representation of the results graphically. - In the text, there is no need to mention the percentages of compliant Journals for all 14 principles, as these are shown in the table. However, the emphasis should be given mainly to the most or least frequent ethical principles not mentioned in the Pediatric Dentistry Journals. | Flowchart for search results added  Results presented as absolute numbers in text  Bar graph for table 2 added  Results presented as absolute numbers in text | Figure 1  Lines 147-167  Figure 2  Lines 157-171 |
| DISCUSSION   - This section is well written, and all aspects are analyzed correctly. - In the second paragraph, reference citation “7” is repeated and a citation is missing at the end of this paragraph. | Reference 7 deleted; reference 8 added at end of this paragraph | Line 211 |
| CONCLUSIONS   - Line 259 🡪Please start with a new sentence “…Ethical issues…” - It would be preferable and more readable if you could present the conclusions using bullets instead of a single paragraph. | Started with desired line  Conclusion presented in bullets | Lines 269-279 |
| TABLES   - Please use the same format for table titles, as follows: “Table 1. This is the Table 1 Title.” | Format corrected | Line 176 |
| REFERENCES   - Please cite references in brackets (for example, “[1]” or “[2-5]” or “[3,7,9]”). - There is inconsistency in the references. Firstly, references with more than six authors should list the first six author names, followed by “et al.” Moreover, all references should follow exactly the same style, | References cited in brackets  Referencing of authors changed as per instructions | Cited throughout the manuscript  References 8, 15, 16 |
| Reviewer #1: The authors have to decide which terminology to use, and apply it throughout the text, is it ethical requirements, guidelines or principles? – submission guidelines as per Ethical Principals | Author submission guidelines- used throughout | Modified throughout the manuscript |
| Title to be revised: Authors submission guidelines, a survey of Pediatric Dentistry journals regarding ethical issues | Title modified | Lines 1 and 2 |
| Aims: Missing from document, currently only in the abstract, thus to be added in the document. | Aims & Objectives added | Lines 73-74 |
| Methods and Materials: How was the checklist compiled, was it based on the ICJME document? Which references were used for? To add at page 6 line 111  Was the checklist/ survey been validated priorly of doing the survey? What was the % of disagreement between the two evaluators? Was disagreement found mostly on one specific or several items of the checklist? | The checklist for marking compliance was mainly used from the previous article of same authors-Mathur VP, Dhillon JK, Kalra G, Sharma A, Mathur R. Survey of instructions to authors in Indian and British Dental Journals with respect to ethical guidelines. J Indian Soc PedodPrev Dent. 2013;31(2):107-112. Modifications were done in context wit recent amendments in ICMJE guidelines- Recommendations for the Conduct, Reporting, Editing, and Publication of Scholarly Work in Medical Journals. http://www.icmje.org/icmje-recommendations.pdf.  reference added line 105-107.  Disagreement between evaluators were not tested as the findings were cross verified by a group of other two authors | lines 108-110 |
| Results Raw results per Journal and per principle to be included either as Descriptive Table or as appendix. Table 1, to add on an extra column the Impact Factor of the Journals, for those that it exists.  Table 2, could be graphically presented in a horizontal bar/ histogram for each item of the checklist, to make results easier to apprehend. | Raw results/ data entry may be shared only for perusal of editors and reviewers only  Added  Figure 2 added | Table 1 |
| Additional analysis: 1) The checklist could be grouped in 3 categories, by weight of contribution and then the results presented accordingly by histogram or pie chart. Checklist categories to be considered are A=1 through 6, B=7-12, C=13,14. This type of analysis will provide some weight on the ethical principles missing per journal. 2) The association of the Impact Factor IF on the ethical principles, to be investigated. | The bar graph has been described which is self explanatory for individual ethical principles in Figure 2  Calculating association is not possible as not all journals have an impact factor, however, it has been mentioned in table 1 |  |
