# Supplementary material for: Authors submission guidelines, a survey of pediatric dentistry journals regarding ethical issues
Source: PLoS One. 2022 Jan 19;17(1):e0261881. doi: 10.1371/journal.pone.0261881 (PMC8769321; doi:10.1371/journal.pone.0261881)
Supplement: Attachment — Submitted filename: PONE-D-21- 18572_R1.docx [file pone.0261881.s004.docx]

**PONE - D – 21 – 18572 _ R1**

- The appropriate sections have been correctly mentioned and subsections in the Materials and Methods section were added.
- In the abstract section, the “background” was correctly deleted and it is now well structured. However, I would recommend to replace “Aim and Objectives” with just “Objective”.
- The significance of the study and the reason of its conduction are now well highlighted in the Introduction section. However, in the Aim and Objective section, it would be preferable if there was no title and just the beginning of the sentence was: “The aim of the present study was to assess the pattern of submission guidelines regarding the ethical issues given to authors in various Pediatric Dental Journals.“
- The subsections of the MATERIALS AND METHODS section should follow the same format. All for example bold and italics.
- Please rephrase lines 85-86: “However, the journals which were discontinued or with irregular publishing were excluded.”
- The inclusion and exclusion criteria are now more clearly presented.
- In line 93, the keyword “Pediatric Dentistry” is mentioned twice.
- In the figure and table legends please delete the phrase: “This is the figure 1…” or “This is the Table 1…”. For Figure 1, you can just write: “Figure 1: Literature Search Methodology and Results.”
- In conclusions, in the second bullet please split the sentence in order to be clearer.
- In conclusions, the following sentence: “There is a need that journals must carefully cover all aspects on ethical conduct and research reporting in submission guidelines for authors.” could be placed in a separate bullet.
- Please check again the references so that all follow the same format. Inconsistencies exist again, so make sure that the dates, the authors etc. are written in the same way.
