# Supplementary material for: Authors submission guidelines, a survey of pediatric dentistry journals regarding ethical issues
Source: PLoS One. 2022 Jan 19;17(1):e0261881. doi: 10.1371/journal.pone.0261881 (PMC8769321; doi:10.1371/journal.pone.0261881)
Supplement: Attachment — Submitted filename: Response to reviewers.docx [file pone.0261881.s005.docx]

| REVIEWER´S COMMENTS | AUTHOR´S REPLY | Changes done on page/line number |
| --- | --- | --- |
| Reviewer #1:  Please state the reasons the raw data of your work cannot be made public as an Appendix to this publication | Raw data has been uploaded as a separate file with main manuscript | A separate document named as Appendix attached |
| REVIEWER # 2:  In the abstract section, the “background” was correctly deleted and it is now well structured. However, I would recommend to replace “Aim and Objectives” with just “Objective”. | Aim and Objectives” changed to “Objective”. | Line 17 |
| REVIEWER # 2:  The significance of the study and the reason of its conduction are now well highlighted in the Introduction section. However, in the Aim and Objective section, it would be preferable if there was no title and just the beginning of the sentence was: “The aim of the present study was to assess the pattern of submission guidelines regarding the ethical issues given to authors in various Pediatric Dental Journals.“ | Changes done | Line 73-74 |
| • The subsections of the MATERIALS AND METHODS section should follow the same format. All for example bold and italics. | Changes done  Sub headings changed in bold and italic | Line 76, 91, 104, 129, 135, 141 |
| • Please rephrase lines 85-86: “However, the journals which were discontinued or with irregular publishingwere excluded.” | Changes done  Language changed | Line 86 |
| • In line 93, the keyword “Pediatric Dentistry” is mentioned twice. | Two different key words used are- Paediatric Dentistry and Pediatric Dentistry due to differences in British and American english ; spellings corrected | Line 93 |
| • In the figure and table legends please delete the phrase: “This is the figure 1…” or “This is the Table 1…”. For Figure 1, you can just write: “Figure 1: Literature Search Methodology and Results.” | Legends for table and figures corrected | Line 172, 176, 181 |
| In conclusions, in the second bullet please split the sentence in order to be clearer. | the second bullet has been split into different sentences as second and third bullet for clarity | Line 271-276 |
| •In conclusions, the following sentence: “There is a need that journals must carefully cover all aspects on ethical conduct and research reporting in submission guidelines for authors.” could be placed in a separate bullet. | Bullet separated for “There is a need that journals must carefully cover all aspects on ethical conduct and research reporting in submission guidelines for authors.” in conclusion | Line 280- 281 |
| •Please check again the references so that all follow the same format. Inconsistencies exist again, so make sure that the dates, the authors etc. are written in the same way. | References checked and modified | Format of References number 1,2,6,7,8,9, 13,14, 16 modified |
