# Supplementary material for: Authors submission guidelines, a survey of pediatric dentistry journals regarding ethical issues
Source: PLoS One. 2022 Jan 19;17(1):e0261881. doi: 10.1371/journal.pone.0261881 (PMC8769321; doi:10.1371/journal.pone.0261881)
Supplement: Attachment — Submitted filename: Response to Reviewers.docx [file pone.0261881.s006.docx]

| Additional Editor Comments | Reply to Comments | Line/Page number |
| --- | --- | --- |
| After close evaluation of the manuscript and the uploaded raw data, I have noticed that the Impact Factor presented for the journals is not correct (both in raw data information and Table 1). It appears that you have mixed- up Impact scores of some journals with impact factors.  For example, European Archives of Paediatric Dentistry has no Impact Factor currently (Clarivate Analytics), but reports an Impact score of 2.16. this is different. pls correct throughout and re- examine closely for all journals. As in others, for example Intern J Paediatr Dent, the correct IF is given. Also, for others there is inconsistency on what it is reported in the Table and Appendix file with raw data.  Please use the Clarivate Analytics of the Web of Science Journal Citation Reports 2020, consistently. | The impact factor values have been modified in the manuscript and appendix as suggested and are highlighted in yellow | Line 154-156  Table 1 in manuscript |
